# Post-COVID-19 dry eye negatively impacts the patient’s quality of life

**DOI:** 10.5935/0004-2749.2024-0112

**Published:** 2024-10-31

**Authors:** Rosalia Antunes-Foschini, Ilen Ferreira Costa, Lívia Pimenta Bonifácio, Eduardo Melani Rocha, André Messias, Valdes Roberto Bollela, Fernando Bellissimo-Rodrigues

**Affiliations:** 1 Department of Ophthalmology, Otorhinolaryngology, and Head and Neck Surgery, Faculdade de Medicina de Ribeirão Preto, Universidade de São Paulo, Ribeirão Preto, SP, Brazil; 3 Department of Social Medicine, Faculdade de Medicina de Ribeirão Preto, Universidade de São Paulo, Ribeirão Preto, SP, Brazil; 4 Department of Internal Medicine, Faculdade de Medicina de Ribeirão Preto, Universidade de São Paulo, Ribeirão Preto, SP, Brazil

**Keywords:** COVID-19, Coronavirus infections, SARS-CoV-2, Eye diseases, Epidemiology, Ocular surface, Public health

## Abstract

**Purpose:**

To describe the ophthalmological findings of dry eye disease and its relation to the quality of life of COVID-19 survivors.

**Methods:**

COVID-19 survivors who had previously been hospitalized at Hospital das Clínicas de Ribeirão Preto complex underwent an ophthalmological evaluation, which included a dry eye disease questionnaire, break-up time, fluorescein staining, and Schirmer test. We collected the presenting and best-corrected visual acuity, sociodemographic data, personal medical history, and scores from a self-reported quality of life questionnaire (WHOQOL-bref). According to the severity of the acute phase of the disease, the patients were classified into mild-to-moderate, severe, and critical groups.

**Results:**

Ninety-five patients (190 eyes) were evaluated 100 ± 44 days after the onset of COVID-19 symptoms. Of these, 83 patients (87.3%) completed the WHOQOL-bref questionnaire. Ten patients (12.0%) had mild-to-moderate COVID-19, 41 (49.4%) had severe COVID-19, and 32 (38.6%) had critical COVID-19. The median best-corrected visual acuity was logMAR 0 (0-1). Approximately 26.3% patients had a history of dry eye disease or severe dry eye symptoms (frequent or constant ocular dryness and irritation). There was an association between the proportion of patients with dry eye disease and the quality of life (p=0.014) and health (p=0.001). Furthermore, there was a significant trend between the proportion of patients with dry eye disease and how they rated their health and quality of life (p=0.0004 and 0.0027, respectively.

**Conclusions:**

There is a significant negative correlation between the proportion of patients with dry eye disease and their self-reported quality of life.

## INTRODUCTION

COVID-19 is a severe acute respiratory syndrome that was declared a pandemic in March 2020 by the World Health Organization^(1)^. Caused by the coronavirus SARS-CoV-2^(2)^, COVID-19 has affected millions of people worldwide, including the Chinese ophthalmologist Dr. Li Wenliang, who was one of the first physicians to warn of its severity and rapid spread^(3)^.

There has been an increase in the signs and symptoms of dry eye disease (DED) in viral infections such as hepatitis C (hepatitis C virus, HCV), diffuse infiltrative lymphocytosis syndrome (human immunodeficiency virus, HIV), herpetic disease (herpes simplex virus-1, HSV-1), infectious mononucleosis (Epstein Barr virus, EBV)^(4^-^6)^, and COVID-19^(7^-^9)^, as well as adult T-cell leukemia/lymphoma and human T-cell lymphotropic virus-1 (HTLV-1)-associated myelopathy which are caused by HTLV-1^(4^,^5)^. Several studies have also demonstrated a correlation between DED and a poor quality of life^(10^-^12)^.

In the present study, we aimed to describe the ophthalmological findings related to DED and its relation to the quality of life and health of COVID-19 survivors.

## METHODS

This cross-sectional study is part of a large cohort study named RECOVIDA, which aimed to comprehensively describe the clinical picture of the post-COVID-19 condition^(7^, ^13)^. Patients were recruited during follow-up in the infectious disease ambulatory care setting after the acute phase of the disease had passed. Most of the patients who presented with a severe or critical disease had been previously hospitalized at the *Hospital das Clínicas de Ribeirão Preto* complex. A small proportion of the patients who presented with a mild-to-moderate disease had not been hospitalized during the acute phase. The patients were classified into the mild-to--moderate (mild symptoms that did not require oxygen support or hospitalization), severe (severe symptoms that required hospitalization and/or oxygen support), or critical (severe symptoms that required hospitalization, intensive care, and intubation or patients who developed specific complications) group, as mentioned in a previous study^(7)^. Data were collected from the patients’ medical records from the time of hospitalization to the day they attended the infectious disease ambulatory setting between March 2020 to March 2021. During this time, the SARS-CoV-2 B lineage was the most commonly sequenced virus at the hospital^(14)^.

Both RECOVIDA and our study were approved by the Comitê de Ética em Pesquisa do Hospital das Clínicas da Faculdade de Medicina de Ribeirão Preto (CAAE: 31,172,720.9.0000.5440, No: 4.000.153, date: 04/30/20; and CAAE: 33,654,820.1.0000.5440, No: 4.103.401, date: 06/22/20). The study was conducted in accordance with the principles of the Declaration of Helsinki, and written informed consent was obtained from all the patients. The participants were not involved in the design, conduct, reporting, or dissemination plans of our study.

An abbreviated version of the World Health Orga-nization’s Quality of Life (WHOQOL-100) questionnaire (WHOQOL-bref; Supplementary material) was used to assess the quality of life. The questionnaire was administered by a single member of the infectious disease team. Subsequently, an ophthalmological examination was performed by an ophthalmologist who did not have access to the questionnaire. The WHOQOL-bref is based on the patient’s perception of their quality of life before and after COVID-19. This questionnaire is divided into the following four domains: physical, psychological, social, and environmental relationships^(15^, ^16)^.

This study included patients who attended the post--COVID-19 ambulatory care setting between March 2020 and March 2021, and the Ophthalmologic ambulatory care setting between July 2020 and March 2021, during the recovery phase of the disease. Of the 135 patients who were contacted, 16 refused to participate and 24 did not attend the appointment. Finally, 95 patients (190 eyes) were examined. The patients were diagnosed on the basis of a positive polymerase chain reaction test result for SARS-CoV-2 that was performed using throat or nasopharynx swab samples.

On the day of the ophthalmological examination, the patients were questioned regarding their ocular signs and symptoms. They responded to a short questionnaire that included the following three items: 1. How often do your eyes feel dry? (0: never, 1: sometimes, 2: often, or 3: constantly); 2. How often do your eyes feel irritated?; and 3. Have you ever been diagnosed (by a clinician) with dry eye syndrome? (1: Yes, 2: No). Patients were considered to have DED if they responded with “often” or “constantly” for questions 1 and 2 or they responded with “yes” for question 3^(7^, ^17^-^19)^. Subsequently, a complete ophthalmological examination was performed, which included presenting and best-corrected visual acuity (BCVA; presented as logMAR), biomicroscopy, and dry eye tests. The dry eye tests were performed according to the Dry Eye Workshop guidelines^(19)^. The break-up time (BUT) was considered positive for dry eye if the break-up time was <7 s in the worse eye. In corneal fluorescein staining, the cornea was divided into five zones (one central and four peripheral zones). Each zone was scored from 0 (no stain) to 3 (great stain), and the total score varied from 0 to 15. The fluorescein staining test was considered positive if the score was 3 or more in at least one eye. The tear flow was measured using the Schirmer test without anesthesia, and the patient was considered to have a dry eye if the worse eye showed ≤5 mm of wetness. We defined DED as a positive response for dry eye in the short questionnaire and at least one positive dry eye test in at least one eye.

We assessed for differences in the signs and symptoms of DED among the mild-to-moderate, severe, and critical groups and between male and female patients. We also evaluated for an association between the WHOQOL-bref data (self-assessment of the quality of life [WHOQOL1] and self-assessment of health [WHOQOL2]) and the following ophthalmological examination findings: presenting visual acuity in the right eye (visual acuity when answering the WHOQOL questionnaire), ocular pain, blurry vision, disease severity during hospitalization, and a positive history of DED or severe dry eye symptoms (frequent or constant ocular dryness and irritation). We also assessed for an association between the WHOQOL-bref questionnaire score and the time interval between COVID-19 onset and WHOQOL-bref administration.

We organized the data using Microsoft Excel (version 16.16.27; Redmont, WA, USA) and performed statistical analyses using Stata (Stata/IC 15.1; StataCorp, College Station, TX, USA). We assessed for Gaussian distribution using the Doornik-Hansen multivariate normality test. We used the one-way ANOVA, Wilcoxon rank-sum (Mann-Whitney), Kruskal-Wallis, and Wilcoxon matched-pairs signed-rank tests to assess the continuous variables. The categorical variables were assessed using the two-sided Fisher’s exact test. The Cochran Armitage test was performed using JMP^®^ (version 16.2.0; Ottawa, CA) to assess the trends between the frequency of DED history or presence of severe dry eye symptoms and the self-reported quality of life and health. A p-value of <0.05 was considered statistically significant.

## RESULTS

Of the 95 patients, 10 (10.5%) had mild-to-moderate COVID-19, 46 (48.4%) had severe COVID-19, and 39 (41.1%) had critical COVID-19. Most of the men (56.3%) had critical COVID-19. The mean duration of hospital stay was 17 ± 14 days. The mean interval between the onset of COVID-19 symptoms and the day of WHOQOL-bref administration was 73 ± 42 days. Furthermore, the mean interval between the onset of COVID-19 symptoms and the day of ophthalmological examination was 100 ± 44 days (range 31-235 days). Thirteen (13.7%) patients were healthcare professionals, 42 (44.2%) patients were obese (body mass index >30), and 21 (22.1%) patients were previously smokers. Of the 95 patients, 44 (46.3%) had systemic arterial hypertension, 36 (37.9%) had diabetes mellitus, and 18 (18.9%) had dyslipidemia. Furthermore, 73 patients (76.8%) were being treated with long-term medications.

Table 1 shows the data related to the WHOQOL-bref questionnaire. Among the four evaluated domains, the physical domain demonstrated the most changes. Only 83 of the 95 patients completed the WHOQOL-bref questionnaire. Of the 83 patients, 28 (33.7%) reported a worsening in their quality of life after COVID-19. Furthermore, there was a statistically significant worsening in the quality of life after COVID-19 (p=0.0003, paired Wilcoxon test).

**Table 1:** WHOQOL-bref^*^ questionnaire scores in the physical, psychological, social, and environmental domains, as well as the self-rated quality of life and health

WHOQOL questionnaire (4-20 points)	Median	Interquartile range
Physical	12.6	11.4-13.7
Psychological	13.3	12.7-15.3
Social	16.0	13.3-17.3
Environmental	14.0	13.0-15.0
Self-rated quality of life	14.3	12.0-16.0
Self-rated quality of health	13.7	12.0-16.0

* The closer to 20, the better the WHOQOL self-assessment.

There was no association between the WHOQOL1 or WHOQOL2 and presenting visual acuity (p≥0.782), blurry vision (p≥0.567), ocular pain (p≥0.506), or disease severity during the acute phase (p≥0.185). Furthermore, there was no association between the WHOQOL1 or WHOQOL2 and the time interval between COVID-19 onset and WHOQOL administration. However, there was a statistically significant association between a history of DED or severe dry eye symptoms and the WHOQOL1 or WHOQOL2 (p=0.014 and p=0.001, respectively; two-tailed Fisher’s exact test) (Table 2). Furthermore, there was a statistically significant association between the proportion of patients with a history of DED or severe dry eye symptoms and how they rated their health (p=0.0004, Cochran Armitage test) and quality of life (p=0.0027, Cochran Armitage test). Thus, the poorer the self-rated health or quality of life, the greater the proportion of individuals with DED or severe dry eye symptoms.

**Table 2:** Proportion of patients with a dry eye disease history or those with severe dry eye symptoms and their self-reported quality of life or health

Classification	Very bad	Bad	Neither bad nor good	Good	Very good	Total	p-value
Quality of life	2/3	3/3	7/23	11/49	0/5	23/83	**0.014** ^a^
Health	2/2	5/12	12/27	3/33	1/9	23/83	**0.001** ^a^

a Two-sided Fisher’s exact test. The numerator indicates the number of patients with DED or dry severe symptoms, and the denominator indicates the total number of patients who answered the questionnaire.

Among the 95 study participants, 4 (4.2%) had a previous history of DED and 21 (22.1%) were newly diagnosed with DED. Table 3 presents the demographic and ocular data of the 95 individuals. The presenting visual acuity and BCVA were significantly different between the three study groups (p≤0.03, Wilcoxon matched-pairs signed-rank test). However, the presence of a dry DED history or the frequency of severe dry eye symptoms did not differ with ocular pain, blurry vision, or disease severity. A history of dry eye and the prevalence of its symptoms was higher in women (n=18/47; 38.3%) than in men (n=7/48; 14.6%) (p=0.011, two-tailed Fisher’s exact test).

**Table 3:** Ocular and demographic data of COVID-19 survivors who were classified according to their disease severity

	Mild-to-moderate (n=10)	Severe (n=46)	Critical (n=39)	p-value
Male/Female (n=95)	3:7	18:28	27:12	0.008^a^
Age, years (n=95)	51.5 ± 8.9	56.9 ± 14.6	54.7 ± 10.7	0.064^b^
Presenting VA RE (n=95)	0.05 (0-0.1)	0 (0-0.2)	0.1 (0-0.2)	0.263^c^
Presenting VA LE (n=95)	0 (0-0)	0 (0-0.2)	0.1 (0-0.2)	0.011^c^
BCVA RE (n=93)	0 (0-0)	0 (0-0)	0 (0-0.2)	0.048^c^
BCVA LE (n=93)	0 (0-0)	0 (0-0.1)	0 (0-0.2)	0.015^c^
Schirmer ≤5 mm in the WE (n = 95)	1 (10.0%)	8 (17.4%)	12 (30.8%)	0.266^a^
BUT <7 s in the WE (n = 95)	1 (10.0%)	7 (15.2%)	3 (7.7%)	0.598^a^
Fluorescein stain ≥3 in the WE (n=95)	1 (10.0%)	8 (17.4%)	4 (10.3%)	0.755^a^
History of dry eye or severe symptoms^φ^ (n = 95)	6 (60.0%)	10 (21.7%)	9 (23.1%)	0.051^a^
Dry eye diagnosis (n=95)	1 (10.0%)	7 (15.2%)	3 (7.7%)	0.598^a^
Blurry vision (n=95)	3 (30.0%)	24 (52.2%)	23 (59.0%)	0.267^a^
Ocular pain (n=95)	1 (10.0%)	4 (8.7%)	5 (12.8%)	0.888^a^

Data are presented as mean ± standard deviation, median (interquartile range), or frequency (%).

VA= visual acuity; RE= right eye; LE= left eye; WE= worse eye; BCVA= best-corrected visual acuity; BUT= break-up time.

φ = Dryness or irritation.

a Fisher test;

b One-way ANOVA;

c Kruskal-Wallis test.

## DISCUSSION

In the present study, we described the DED-related data in COVID-19 survivors and their association with the WHOQOL-bref. Of the 95 study participants, only 4 (4.2%) had a previous history of DED. Furthermore, the prevalence of a history of DED or severe dry eye symptoms was higher in our study (prior DED diagnosis, n=4, 4.2%; newly diagnosed DED, n=21, 22.1%; total, n=25, 26.3%) than in a Brazilian study that used the same approach (three questions regarding ocular signs and symptoms) prior to the COVID pandemic. They observed a prevalence of 13.2% in a population aged 40-60 years^(18)^. Our frequency is also higher than the overall prevalence of dry eye (12.8%)^(18)^. Dry eye has been described in numerous viral infections^(4^-^6)^, including COVID-19. The increased frequency of severe dry eye symptoms in COVID-19 may be attributed to the constant use of masks, leading to more intense dry eye symptoms^(20)^ and signs^(21^,^22)^, such as worsening Schirmer test patterns, BUT, and fluorescein staining. Another hypothesis is that people with ocular surface disease may be more susceptible to SARS-CoV-2 because their seroprevalence for COVID-19 is higher than those without ocular surface disease^(9)^. In another study similar to ours, a higher frequency of dry eye was observed in COVID-19 survivors than in controls^(8)^. Another possible explanation to the increased frequency of dry eye in our sample may be that SARS-CoV-2 trigger an autoimmune response, similar to other viral infections^(4^-^6)^, which may increase the incidence of Sjögren’s Syndrome^(23)^. However, the underlying mechanisms and the relationship between viral infections and autoimmune diseases remain unknown. Further studies are required to clarify this relationship and the underlying mechanisms to provide a better approach for managing these conditions.

In our study, a higher proportion of patients with a history of DED or severe dry eye symptoms self-reported a poor quality of life. This result is consistent with that of other studies during^(21)^ and before^(10)^ the COVID-19 pandemic. Previous studies have demonstrated that DED negatively impact the quality of life. Li et al.^(11)^ observed an association between decreased quality of life and increased ocular symptoms when comparing patients with DED with healthy controls. In a study on Korean women, DED negatively impacted the quality of life and was associated with pain/discomfort and depression/anxiety^(12)^.

In our study, we also found that the poorer the WHOQOL, the greater the proportion of patients with a history of DED or severe dry eye symptoms. This may be attributed to the fact that the cornea is the most sensitive structure in the body. Therefore, any changes on its surface would produce severe symptoms that could lead to a rapid decline in the quality of life and health.^(24^,^25)^ However, we could not identify a causal relationship between these two variables (DED preceding quality of life and health low scores) as they were assessed independently. Furthermore, we did not specifically ask the patients whether the dry eye affected their quality of life. Thus, this association may only be fortuitous.

Our study has some limitations. There may have been a selection bias as participants were more likely to join the study if they had ophthalmologic symptoms. Second, we did not control our data for climate factors and patient medications or occupation, which may have influenced the results related to dry eye signs and symptoms. The ocular surface findings did not correlate with the clinical severity of COVID-19. The main reason for this may be the low predictive value of ocular surface and dry eye tests isolated^(26)^ and the individual compensatory mechanisms of tissue damage revealed in these patients. Our data was skewed toward patients with severe and critical COVID, which might explain the higher frequency of DED in this specific group. Furthermore, we do not know the pre-COVID status of the patients’ severe dry eye symptoms or the exact time of symptom onset. So, further studies are needed to elucidate if this association fortuitous or if there is a causal relationship between the exposure (DED after COVID-19) and the event (self-assessed quality of life and health).

In conclusion, 26.3% of COVID-19 survivors presented severe dry eye symptoms or had a history of DED. This is higher than the prevalence in previous studies (4.2%). The physical domain was the most affected on the quality of life questionnaire. Furthermore, we observed that the presence of a DED history or severe dry eye symptoms negatively impacted the self-reported quality of life and health.

## Supplementary material - WHOQOL - VERSÃO EM PORTUGUÊS

### Perguntas sobre qualidade de vida

Instruções

Este questionário é sobre como você se sente a respeito de sua qualidade de vida, saúde e outras áreas de sua vida. Por favor, responda a todas as questões. Se você não tem certeza sobre que resposta dar em uma questão, por favor, escolha entre as alternativas que a lhe parece mais apropriada. Esta, muitas vezes, poderá ser sua primeira escolha.

Por favor, tenha em mente seus valores, aspirações, prazeres e preocupações. Nós estamos perguntando o que você acha de sua vida, tomando como referência as duas últimas semanas. Por exemplo, pensando nas últimas duas semanas, uma questão poderia ser:

**Table:**

	Nada	Muito pouco	Médio	Muito	Completamente
Você recebe dos outros o apoio que necessita?	1	2	3	4	5

Você deve circular o número que melhor corresponde ao quanto você recebe dos outros o apoio de que necessita nestas últimas duas semanas.

Portanto, você deve circular o número 4 se você recebeu “muito” apoio como abaixo.

**Table:**

	Nada	Muito pouco	Médio	Muito	Completamente
Você recebe dos outros o apoio que necessita?	1	2	3	4	5

Você deve circular o número 1 se você não recebeu “nada” de apoio.

Por favor, leia cada questão, veja o que você acha e circule no número que lhe parece a melhor resposta.

**Table:**

	Muito ruim	ruim	Nem ruim nem boa	Boa	Muito Boa
1. Como você avaliaria sua qualidade de vida?	1	2	3	4	5
	**Muito insatisfeito**	**Insatisfeito**	**Nem satisfeito, nem insatisfeito**	**Satisfeito**	**Muito satisfeito**
2. Quão satisfeito(a) você está com a sua saúde?	1	2	3	4	5

As questões seguintes são sobre o quanto você tem sentido algumas coisas nas últimas duas semanas.

**Table:**

	Nada	Muito pouco	Mais ou menos	Bastante	Extremamente
3. Em que medida você acha que sua dor (física) impede você de fazer o que você precisa?	1	2	3	4	5
4. O quanto você precisa de algum tratamento médico para levar sua vida diária?	1	2	3	4	5
5. O quanto você aproveita a vida?	1	2	3	4	5
6. Em que medida você acha que a sua vida tem sentido?	1	2	3	4	5
7. O quanto você consegue se concentrar?	1	2	3	4	5
8. Quão seguro(a) você se sente em sua vida diária?	1	2	3	4	5
9. Quão saudável é o seu ambiente físico (clima, barulho, poluição, atrativos)?	1	2	3	4	5

As questões seguintes perguntam sobre quão completamente você tem sentido ou é capaz de fazer certas coisas nestas últimas duas semanas.

**Table:**

	Nada	Muito pouco	Médio	Muito	Completamente
10. Você tem energia suficiente para seu dia-a-dia?	1	2	3	4	5
11. Você é capaz de aceitar sua aparência física?	1	2	3	4	5
12. Você tem dinheiro suficiente para satisfazer suas necessidades?	1	2	3	4	5
13. Quão disponíveis para você estão as informações que precisa no seu dia-a-dia?	1	2	3	4	5
14. Em que medida você tem oportunidades de atividade de lazer?	1	2	3	4	5

As questões seguintes perguntam sobre quão bem ou satisfeito você se sentiu a respeito de vários aspectos de sua vida nas últimas duas semanas.

**Table:**

	Muito ruim	ruim	Nem ruim nem bom	Bom	Muito Bom
15. Quão bem você é capaz de se locomover?	1	2	3	4	5
	**Muito insatisfeito**	**Insatisfeito**	**Nem satisfeito, nem insatisfeito**	**Satisfeito**	**Muito satisfeito**
16. Quão satisfeito(a) você está com o seu sono?	1	2	3	4	5
17. Quão satisfeito(a) você está com sua capacidade de desempenhar as atividades do seu dia-a-dia?	1	2	3	4	5
18. Quão satisfeito(a) você está com sua capacidade para o trabalho?	1	2	3	4	5
19. Quão satisfeito(a) você está consigo mesmo?	1	2	3	4	5
20. Quão satisfeito(a) você está com suas relações pessoais (amigos, parentes, conhecidos, colegas)?	1	2	3	4	5
21. Quão satisfeito(a) você está com sua vida sexual?	1	2	3	4	5
22. Quão satisfeito(a) você está com o apoio que você recebe de seus amigos?	1	2	3	4	5
23. Quão satisfeito(a) você está com as condições do local onde mora?	1	2	3	4	5
24. Quão satisfeito(a) você está com o seu acesso aos serviços de saúde?	1	2	3	4	5
25. Quão satisfeito(a) você está com o seu meio de transporte?	1	2	3	4	5

A questão seguinte refere-se a com que frequência você sentiu ou experimentou certas coisas nas últimas duas semanas.

**Table:**

	Nunca	Algumas vezes	Frequentemente	Muito frequentemente	Sempre
26. Com que frequência você tem sentimentos negativos tais como mau-humor, desespero, ansiedade, depressão?	1	2	3	4	5

Alguém lhe ajudou a preencher este questionário?___________________________________________________________

Quanto tempo você levou para preencher este questionário?___________________________________________________

**Fonte:** Adapatado de: Fleck MP, Louzada S, Xavier M, Chachamovich E, Vieira G, Santos L, et al. [Application of the Portuguese version of the abbreviated instrument of quality life WHOQOL-bref]. Rev Saude Publica. 2000;34(2):178-83^(15^,^16)^.

## References

[1] Cucinotta D, Vanelli M. (2020). WHO declares COVID-19 a pandemic. Acta Biomed.

[2] Almazroa A, Alamri S, Alabdulkader B, Alkozi H, Khan A, Alghamdi W. (2022). Ocular transmission and manifestation for coronavirus disease: a
systematic review. Int Health.

[3] Petersen E, Hui D, Hamer DH, Blumberg L, Madoff LC, Pollack M (2020). Li Wenliang, a face to the frontline healthcare worker. The first
doctor to notify the emergence of the SARS-CoV-2, (COVID-19),
outbreak. Int J Infect Dis.

[4] Alves M, Angerami RN, Rocha EM. (2013). Dry eye disease caused by viral infection: review
[review]. Arq Bras Oftalmol.

[5] Sipsas NV, Gamaletsou MN, Moutsopoulos HM. (2011). Is Sjögren’s syndrome a retroviral
disease?. Arthritis Res Ther.

[6] Rajalakshmy AR, Malathi J, Madhavan HN, Bhaskar S, Iyer GK. (2015). Patients with dry eye without hepatitis C virus infection possess
the viral RNA in their tears. Cornea.

[7] Costa IF, Bonifácio LP, Bellissimo-Rodrigues F, Rocha EM, Jorge R, Bollela VR (2021). Ocular findings among patients surviving COVID-19. Sci Rep.

[8] Gambini G, Savastano MC, Savastano A, De Vico U, Crincoli E, Cozzupoli GM (2021). Ocular surface impairment after coronavirus disease 2019: a
cohort study. Cornea.

[9] Li S, Qiu Y, Tang L, Wang Z, Cao W, Zhou X (2021). Association of ocular surface diseases with sars-cov-2 infection
in six districts of china: an observational cohort study. Front Immunol.

[10] Uchino M, Schaumberg DA. (2013). Dry eye disease: impact on quality of life and
vision. Curr Ophthalmol Rep.

[11] Li M, Gong L, Chapin WJ, Zhu M. (2012). Assessment of vision-related quality of life in dry eye
patients. Invest Ophthalmol Vis Sci.

[12] Na KS, Han K, Park YG, Na C, Joo CK. (2015). Depression, stress, quality of life, and dry eye disease in
korean women: a population-based study. Cornea.

[13] Bonifácio LP, Csizmar VN, Barbosa-Júnior F, Pereira AP, Koenigkam--Santos M, Wada DT (2022). Long-Term Symptoms among COVID-19 Survivors in Prospective Cohort
Study, Brazil. Emerg Infect Dis.

[14] de Carvalho FS, Slack SD, Barbosa-Júnior F, de Campos MR, Castro GS, Baroni S (2023). Genomic epidemiology of SARS-CoV-2 in large university hospital
cohort: the UnCoVER-Brazil project. Epidemiol Infect.

[15] The WHOQOL Group (1995). The World Health Organization Quality of Life assessment
(WHOQOL): position paper from the World Health Organization. Soc Sci Med.

[16] Fleck MP, Louzada S, Xavier M, Chachamovich E, Vieira G, Santos L (2000). [Application of the Portuguese version of the abbreviated
instrument of quality life WHOQOL-bref]. Rev Saude Publica.

[17] Nalbandian A, Sehgal K, Gupta A, Madhavan MV, McGroder C, Stevens JS (2021). Post-acute COVID-19 syndrome. Nat Med.

[18] Castro JS, Selegatto IB, Castro RS, Miranda EC, de Vasconcelos JP, de Carvalho KM (2018). Prevalence and Risk Factors of self-reported dry eye in Brazil
using a short symptom questionnaire. Sci Rep.

[19] Castro JS, Selegatto IB, Castro RS, Vasconcelos JP, Arieta CE, Alves M. (2017). Translation and validation of the Portuguese version of a dry eye
disease symptom questionnaire. Arq Bras Oftalmol.

[20] Boccardo L. (2022). Self-reported symptoms of mask-associated dry eye: A survey study
of 3,605 people. Cont Lens Anterior Eye.

[21] Mastropasqua L, Lanzini M, Brescia L, D’Aloisio R, Nubile M, Ciancaglini M (2021). Face mask-related ocular surface modifications during COVID-19
pandemic: a Clinical, in vivo confocal microscopy, and immune-cytology
study. Transl Vis Sci Technol.

[22] Bilici S, Toprak A, Buyukuysal C, Ugurbas SH. (2022). The effect of day-long mask wearing on non-invasive break-up
time. Graefes Arch Clin Exp Ophthalmol.

[23] Martelli H, Gueiros LA, de Lucena EG, Coletta RD. (2022). Increase in the number of Sjögren’s syndrome cases in
Brazil in the COVID-19 Era. Oral Dis.

[24] Bonini S, Rama P, Olzi D, Lambiase A. (2003). Neurotrophic keratitis. Eye (Lond).

[25] Belmonte C. (2019). Pain, Dryness, and Itch Sensations in Eye Surface Disorders Are
Defined By a Balance Between Inflammation and Sensory Nerve
Injury. Cornea.

[26] Alves M, Reinach PS, Paula JS, Vellasco e Cruz AA, Bachette L, Faustino J (2014). Comparison of diagnostic tests in distinct well-defined
conditions related to dry eye disease. PLoS One.

